# A Supernumerary Tooth above the Superior Third Molar

**Published:** 1884-06

**Authors:** Geo. H. Harrington


### A Supernumerary Tooth Above the Superior Third Molar.

BY DR. GEO. H. HARRINGTON.

Mr. J. W. C., aged twenty-eight years, has for the past two and a half years been troubled, from time to time, with pain in the right superior, third molar. The tooth was not decayed, except a slight cavity in the center of the crown, not extensive enough to cause pain; upon examination found no soreness, nor anything to account for the pain, which had occurred at intervals for more than two years, often quite severe. Mastication upon it would cause pain. Under percussion the tooth was not sore, save occasionally a slight uneasiness. Soon after the beginning of the difficulty the patient called for an examination. No conclusion was attained as to the cause of the difficulty. He subsequently called; at first at long intervals, but afterward more frequently, and recently every few days. The other teeth were all in good condition, so far as decay was concerned. Some salivary calculus was found upon the teeth; this was removed. Neither of these could have caused the difficulty referred to. Some eighteen months after the occurrence of this affection the eye of the same side became affected, and was treated by an oculist but with very little success; occasionally temporary relief would be obtained. The eye became almost blind. So severe did the pain become in the tooth referred to, that its removal was decided upon. After its removal, in the socket was found a small, hard, protruding substance, covered, however, with a layer of soft tissue. This substance was in the space between the roots of the tooth. The soft substance was removed, and the hard body extracted, which proved to be a supernumerary tooth completely formed.

The inner sides of two roots, the palatine and the anterior buccal, were somewhat absorbed, the former so much so that the pulp canal was quite open. The opening of this, and the encroachment upon the pulp tissue was the occasion of the increasing pain, as this tissue was still living when the tooth was extracted.

As an interesting feature in this case, the palatine root was about one-half absorbed; the greatest absorption was near its juncture with the body of the tooth. The absorption of the anterior buccal root was also near its juncture with the body of the tooth, but less in extent than that of the palatine root.

Another marked feature in this case was the affection of the eye, which was wholly dependant upon the affection in the tooth, as was shown by the fact that within a few days after the removal of the tooth there was an entire recovery of the eye, and that, too, without any local treatment.

This case, considered as a whole, is a very interesting one. It is interesting, because of the location of the supernumerary tooth, and also because of the absorption and exposure of the pulp; interesting because of the peculiar opening, and especially interesting in the influence it exercised upon the eye.

While the tooth remained in the mouth the cause of the difficulty was very obscure; and in this case it could only be ascertained upon removal of the tooth, and it is certain that no local treatment of the eye would have restored it while the tooth remained.

